# Tenecteplase With Concomitant Anticoagulation for Acute Respiratory Failure in Patients With COVID-19: A Randomized Controlled Trial

**DOI:** 10.7759/cureus.54298

**Published:** 2024-02-16

**Authors:** Hooman Poor, Kurt Yaeger, Serina Deeba, Sydney Edwards, Emily Chapman, Xinyan Liu, Elliot Eisenberg, Thomas M Tolbert, Aaron Shpiner, J Mocco

**Affiliations:** 1 Pulmonary, Critical Care, and Sleep Medicine, Icahn School of Medicine at Mount Sinai, New York, USA; 2 Neurological Surgery, Icahn School of Medicine at Mount Sinai, New York, USA

**Keywords:** respiratory failure, thrombolysis, tenecteplase (tnk), covid-19, ards (acute respiratory distress syndrome)

## Abstract

Background

Pulmonary thrombosis and thromboembolism play a significant role in the physiologic derangements seen in COVID-19 acute respiratory failure. The effect of thrombolysis with tenecteplase on patient outcomes is unknown.

Methods

We conducted a randomized, controlled, double-blind, phase II trial comparing tenecteplase versus placebo in patients with COVID-19 acute respiratory failure (NCT04505592). Patients with COVID-19 acute respiratory failure were randomized to tenecteplase 0.25 mg/kg or placebo in a 2:1 proportion. Both groups received therapeutic heparin for at least 72 hours.

Results

Thirteen patients were included in the trial. Eight patients were randomized to tenecteplase and five were randomized to placebo. At 28 days, 63% (n = 5) of patients assigned to the treatment group were alive and free from respiratory failure compared to 40% (n = 2) in the placebo arm (p = 0.43). Mortality at 28 days was 25% (n = 2) in the treatment arm and 20% (n = 1) in the control arm (p = 1.0). No patients in the treatment arm developed renal failure by 28 days compared to 60% (n = 3) in the placebo arm (p = 0.07). Major bleeding occurred in 25% (n = 2) of the treatment arm and 20% (n = 1) in the placebo arm; however, no patients in either arm experienced intracranial hemorrhage.

Conclusions

Tenecteplase with concomitant heparin may improve patient outcomes in patients with COVID-19 respiratory failure. As this study was limited by a small sample size, larger confirmatory studies are needed.

## Introduction

Pathologic specimens from acute respiratory distress syndrome (ARDS) secondary to COVID-19 infection have demonstrated high rates of pulmonary microthrombosis [1,2]. For many patients with COVID-19 ARDS, particularly early in the disease course, hypoxemia is out of proportion to the impairment in lung compliance [3], and venous admixture is unrelated to the fraction of non-aerated lung tissue [4], indicating that COVID-19 ARDS may have distinguishing features from classical ARDS. This dissociation between gas exchange and lung mechanics may be explained by pulmonary microthrombosis [5]. COVID-19 is also associated with a hyperinflammatory and hypercoagulable state [6], resulting in various thromboembolic complications, including pulmonary embolism and ischemic stroke [7,8]. These attributes of COVID-19 make it a potential target for anticoagulant and thrombolytic therapeutic approaches.

Studies on therapeutic anticoagulation for non-critically ill patients with COVID-19 pneumonia have demonstrated improved outcomes [9-11], but therapeutic anticoagulation for critically ill COVID-19 patients has shown no benefit [12], suggesting that anticoagulation alone may not be sufficient once significant pulmonary microthrombosis has occurred and that fibrinolytic therapy may be necessary for more advanced cases as low fibrinolysis is a feature of ARDS [13].

Several case series have demonstrated rapid improvement in gas exchange and/or hemodynamics in patients with COVID-19 ARDS after infusion of tissue plasminogen activator (tPA) [14,15]. A randomized controlled trial of tPA in patients with COVID-19 ARDS requiring mechanical ventilation demonstrated improvements in gas exchange without any episodes of major bleeding; however, these physiologic effects were transient [16], indicating the need for a treatment that provides a more sustained physiologic response.

We conducted a randomized, double-blind, placebo-controlled trial of tenecteplase with concomitant anticoagulation with heparin in patients with early COVID-19 respiratory failure and elevated D-dimer levels to determine if this treatment would improve outcomes in these patients compared to therapeutic anticoagulation alone (ClinicalTrials.gov identifier: NCT04505592). Our hypothesis posited that thrombolysis with tenecteplase in conjunction with concomitant anticoagulation with heparin would improve clinical outcomes in patients with early COVID-19 ARDS compared to therapeutic anticoagulation alone.

This article was previously presented as an abstract at the 2022 American Thoracic Society COVID Across the Care Continuum.

## Materials and methods

Study design

This study was a randomized, double-blind, placebo-controlled trial of tenecteplase with concomitant therapeutic anticoagulation with heparin versus therapeutic anticoagulation alone.

Setting and demographics

Patients were recruited in a tertiary academic hospital in New York City. Consenting patients were included in the trial regardless of sex, race, or ethnicity. Demographic information, including age and gender, was collected via chart review.

Inclusion and exclusion criteria

Eligible patients were 18 to 75 years of age; had polymerase chain reaction-confirmed infection with SARS-CoV-2 virus within 14 days; had respiratory failure secondary to COVID-19 requiring mechanical ventilation for no greater than 24 hours, or high-flow nasal cannula (HFNC), non-rebreather (NRB) mask, or non-invasive positive pressure ventilation (NIPPV) for less than 48 hours; had elevated D-dimer greater than six times the upper limit of normal within the past 72 hours; and patients who were intubated greater than 12 hours prior to randomization or with any evidence of neurologic deficit, with a head CT within 12 hours demonstrating no evidence of acute or subacute infarct or hemorrhage.

Exclusion criteria included current participation in another investigational study within the prior seven days; known hypersensitivity to any ingredients of tenecteplase; active internal bleeding; known bleeding diathesis; use of one of new oral anticoagulants within the last 24 hours (dabigatran, rivaroxaban, apixaban, edoxaban); treatment with a thrombolytic within the last three months prior to randomization (exception for the use of Cathflo® alteplase for occlusions of central venous catheters); baseline platelet count < 80,000/L; baseline blood glucose > 400 mg/dL or < 50 mg/dL; intracranial or intraspinal surgery or trauma within two months; other, non-COVID-19 related, serious, advanced, or terminal illness or life expectancy of less than six months; history of acute ischemic stroke in the last 90 days; history of intracranial bleeding, including hemorrhagic stroke; presumed septic embolus or suspicion of bacterial endocarditis; mechanical ventilation > 48 hours, HFNC, NRB, or NIPPV, or any combination for greater than 96 hours; mechanical ventilation, HFNC, NRB, or NIPPV within the prior 30 days (excluding 48 hours prior to randomization); moribund status suggesting imminent vascular collapse and inability to survive > 72 hours; uncontrolled hypertension defined as systolic blood pressure > 180 mm Hg and/or diastolic blood pressure > 110 mm Hg; age > 75 years; history of traumatic brain injury within two months; recent head trauma with fracture of brain injury; history of heparin-induced thrombocytopenia and/or other hereditary or acquired hemorrhagic diathesis or coagulation factor deficiency; international normalized ratio (INR) > 2 or recent oral anticoagulation therapy with INR > 1.7; pregnancy or lactation within the prior 30 days; chronic liver disease greater than Child-Pugh class B; history of atrial fibrillation, mitral stenosis, left heart thrombosis, or any other condition that precludes administration of tenecteplase or poses a significant hazard to the patient who receives tenecteplase.

Ethics approval

The trial was performed in compliance with the Food and Drug Administration Investigational New Drug regulations (identifier: 150888), was approved by the Institutional Review Board at the Mount Sinai School of Medicine (approval: 20-00894), and was registered with ClinicalTrials.gov (identifier: NCT04505592). Informed consent for trial participation was obtained from the patient, or in the case of incapacitation, the patient’s legally authorized representative.

Randomization

Patients were randomized to tenecteplase or placebo in a 2:1 fashion using the randomization module integrated within the study’s electronic data capture system (REDCap, Vanderbilt University, Nashville, TN). The data and statistics team defined the randomization allocation sequence during the creation and development of the trial database, and this sequence was concealed from investigators and other study staff. Clinicians were blinded to the trial-group assignment for each enrolled patient.

Procedures

Patients were administered tenecteplase 0.25 mg/kg (maximum dose of 25 mg) or placebo. For patients already on therapeutic heparin, heparin infusion was continued. For patients not already on heparin or other anticoagulation, heparin was given with a bolus dose of 60 units/kg once (maximum dose of 5000 units), followed by 12 units/kg/hour. For patients who were receiving enoxaparin at the time of inclusion, heparin was delayed to 12 hours after the most recent enoxaparin dose, beginning at an infusion rate of 12 units/kg/hour without a bolus dose. Partial thromboplastin time (PTT) was checked every six hours, and the heparin infusion was adjusted to achieve a target PTT between 2 and 2.5 times the upper limit of normal. Patients were continued with unfractionated heparin infusion for 72 hours and after that time could be transitioned to low molecular weight heparin at the discretion of the treating physician.

Outcomes

The primary efficacy outcome was the proportion of patients alive and free of respiratory failure (defined as not requiring NRB, HFNC, NIPPV, or mechanical ventilation) at 28 days. Secondary efficacy outcomes included 14-day in-hospital mortality, 28-day mortality, ventilator-free days, respiratory failure-free days (not requiring NRB, HFNC, NIPPV, or mechanical ventilation), vasopressor-free days, vasopressor requirement at 24 and 72 hours, partial pressure of oxygen (PaO2)/fraction of inspired oxygen (FiO2) ratio at 24 hours and 72 hours, ICU-free days, hospital length of stay, new renal failure, and need for renal replacement therapy. New renal failure was defined as a doubling or more of the serum creatinine. The primary safety outcome was the occurrence of intracranial bleeding or major bleeding, defined as a bleeding episode leading to hemodynamic compromise requiring emergency intervention within 72 hours of randomization.

Statistical analysis

The original statistical plan was to enroll 60 participants, a number which was not achieved because of recruitment difficulties. Chi-square and Wilcoxon tests were performed using R statistical software to compare the outcomes in and between the two groups.

Funding

The funder of this investigator-initiated study (Genentech, Inc.) had no role in the study design, data collection, analysis, interpretation, or manuscript preparation.

## Results

**Figure 1:**
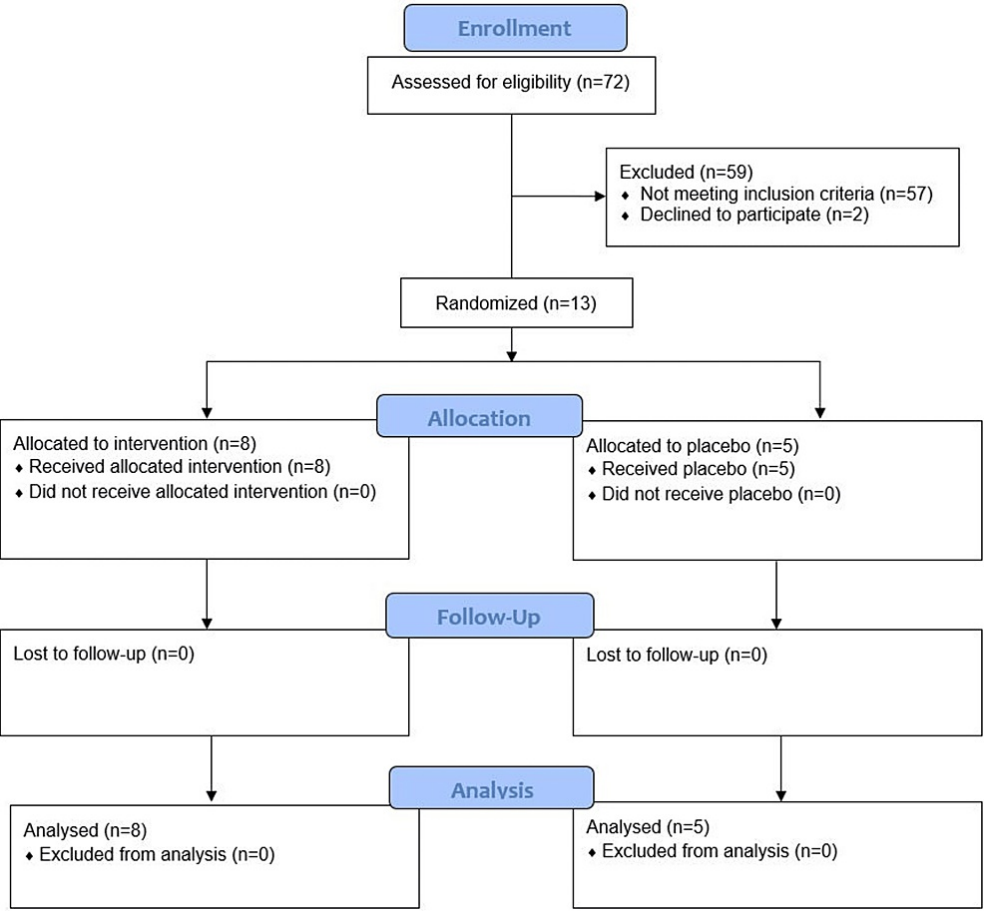
Study flow

From September 2020 to March 2021, 13 patients were enrolled in the study and completed the trial (Figure 1). The trial was terminated early given low enrollment rates. At randomization, six patients required HFNC, five required NIPPV, one required NRB, and one required mechanical ventilation. Table 1 summarizes the baseline characteristics of the patients included in the trial, including demographic information as well as medical comorbidities and presentation data. Of the 13 patients, eight were randomized to tenecteplase, and five were randomized to placebo.

**Table 1:** Baseline characteristics IQR: interquartile range; BMI: body mass index; COPD: chronic obstructive pulmonary disease; HFNC: high-flow nasal cannula; NRB: non-rebreather; NIPPV: non-invasive positive pressure ventilation.

Variable	Control group	Tenecteplase group
Number of patients, n (%)	5 (38%)	8 (62%)
Age, years, median (IQR)	71 (52-73)	67.5 (55-71)
Male sex, n (%)	2 (40%)	7 (88%)
BMI, kg/m^2^, median (IQR)	26 (24-27)	35 (31-39)
Comorbidities, n (%)		
Hypertension	3 (60%)	6 (75%)
Diabetes	1 (20%)	2 (25%)
Coronary artery disease	0 (0%)	3 (38%)
Obstructive sleep apnea	0 (0%)	1 (13%)
Congestive heart failure	0 (0%)	0 (0%)
COPD	0 (0%)	0 (0%)
Asthma	0 (0%)	0 (0%)
Interstitial lung disease	0 (0%)	0 (0%)
Pulmonary hypertension	0 (0%)	0 (0%)
Chronic kidney disease	0 (0%)	0 (0%)
Cerebrovascular accident	0 (0%)	0 (0%)
Venous thromboembolism	0 (0%)	0 (0%)
Respiratory support, n (%)		
Mechanical ventilation	0 (0%)	1 (13%)
HFNC	2 (40%)	4 (50%)
NRB	0 (0%)	1 (13%)
NIPPV	3 (60%)	2 (25%)
Steroids, n (%)	5 (100%)	8 (100%)
Remdesivir, n (%)	2 (40%)	7 (88%)
P/F ratio, median (IQR)	87 (72-131)	80 (67-111)
Creatinine, mg/dL, median (IQR)	0.81 (0.48-1.14)	0.86 (0.73-1.06)

At 28 days, 63% (n = 5) in the treatment arm were alive and free from respiratory failure compared to 40% (n = 2) in the placebo arm (p = 0.43). Mortality at 28 days was 25% (n = 2) in the treatment arm and 20% (n = 1) in the control arm (p = 1.0). There was no difference in the P/F ratio between the two groups at 24 or 72 hours. No patients in the treatment arm developed renal failure by 28 days compared to 60% (n = 3) in the placebo arm (p = 0.07). There was also no difference between the two groups with respect to ventilator-free and respiratory failure-free days, ICU-free days, and hospital length of stay. Major bleeding occurred in 25% (n = 2) of the treatment arm and 20% (n = 1) of the placebo arm (all of which was gastrointestinal bleeding); however, no patients in either arm experienced intracranial hemorrhage. None of the participants who died in the study died as a result of bleeding complications. Table 2 summarizes outcome data for the entire group as well as the treatment and placebo groups individually.

**Table 2:** Outcomes IQR: interquartile range; ICU: intensive care unit.

Variable	Total	Tenecteplase	Placebo	p-value
Number of patients	13	8	5	-
Alive and free from respiratory failure at 28 days, n (%)	7 (54%)	5 (63%)	2 (40%)	0.42
14-day mortality, n (%)	3 (23%)	2 (25%)	1 (20%)	1.00
28-day mortality, n (%)	3 (23%)	2 (25%)	1 (20%)	1.00
Ventilator-free days, median (IQR)	19 (4-28)	18 (3-28)	19 (9-28)	0.70
Respiratory failure-free days, median (IQR)	1 (0-5)	0.5 (0-3)	3 (1-5)	0.45
Vasopressor-free days, median (IQR)	9 (8-18)	9 (4.5-18.5)	9 (9-18)	0.78
P/F ratio at 24 hours, median (IQR)	97 (71-114)	89 (68-111)	97 (89-146)	0.31
P/F ratio at 72 hours, median (IQR)	85 (73-95)	89 (63-91)	78 (77-137)	0.62
New renal failure, n (%)	3 (23%)	0 (0%)	3 (60%)	0.07
Hospital length of stay, days, median (IQR)	12 (10-29)	11 (7.8-29)	13 (12-29)	0.41
ICU-free days, median (IQR)	1 (1-6)	1 (1-7)	1 (1-5)	0.74
Major bleeding, n (%)	3 (23%)	2 (25%)	1 (20%)	1.00

## Discussion

While contemporary research has demonstrated that pulmonary microthrombosis plays a substantial role in the pathophysiology of COVID-19 respiratory failure [1,2], optimal targeted treatment for pulmonary microthrombosis in the setting of COVID-19 respiratory failure is less clear. Although therapeutic anticoagulation alone has been shown to be ineffective among critically ill patients with COVID-19 respiratory failure [12], it is possible that for these patients, the disease and thrombotic burden have progressed too far for anticoagulation alone to be effective, and that thrombolysis may be required. In our study, concomitant anticoagulation was used with thrombolysis to prevent immediate rethrombosis [14]. Tenecteplase was chosen as the thrombolytic agent as alteplase is contraindicated for concomitant use with heparin infusion. Tenecteplase, a modified form of alteplase, is safe to administer with concomitant heparin infusion and may be uniquely suited to be a valuable thrombolytic agent for patients with COVID-19 ARDS [17]. Tenecteplase binds more selectively to fibrin and has a longer half-life than alteplase, allowing it to be administered as a single bolus rather than a continuous infusion [18]. Compared to alteplase, tenecteplase also has increased resistance to PAI-1, a plasminogen activator inhibitor that plays a role in fibrinolytic activity and has been demonstrated to be increased in patients infected with COVID-19 compared to healthy controls and those with other forms of respiratory infection [13]. A previous investigation revealed that increased PAI-1 levels may play a role in the level of thrombosis and fibrin deposition exhibited in patients infected with COVID-19 [18], providing a possible therapeutic target for the development of COVID-19 treatments and interventions. The ability of tenecteplase to resist PAI-1 makes it a promising candidate for improving outcomes in patients with COVID-19 respiratory failure by promoting fibrinolysis.

While the primary endpoint was not met, this trial did, however, yield intriguing results in other physiological domains. Of note, while underpowered for statistical confirmation, there was a trend toward improvement in the rate of patients alive and free from respiratory failure for the treated cohort versus the placebo cohort, suggesting a possible signal toward benefit. Furthermore, there was a trend toward decreased renal failure in patients treated with tenecteplase compared to placebo, indicating a possible connection between administration of tenecteplase with concomitant anticoagulation and preserved renal function in patients with COVID-19 respiratory failure. This finding presents an opportunity for the development of future investigations aimed at understanding and preserving renal function in patients with COVID-19 respiratory failure and other prothrombotic conditions. Additionally, although major bleeding occurred in both groups, no patients in either arm experienced intracranial hemorrhage, a positive indicator of tenecteplase’s safety for clinical use and future trials.

This trial was limited by the small sample size and the use of therapeutic anticoagulation in the control arm as the results of clinical studies demonstrating harm with therapeutic anticoagulation in critically ill COVID-19 patients were published after the completion of this study. Further investigation into the effectiveness of this treatment approach with a larger sample size may generate more conclusive results and provide additional therapy in the armamentarium for patients with COVID-19 respiratory failure.

## Conclusions

While significantly underpowered to confirm the primary outcome of being alive and free of respiratory failure in patients with COVID-19 respiratory failure, this study provides a promising signal that the use of tenecteplase with concomitant therapeutic anticoagulation may facilitate improved outcomes in certain patients experiencing respiratory failure from COVID-19.
